# Human motion characteristics in relation to feeling familiar or frightened during an announced short interaction with a proactive humanoid

**DOI:** 10.3389/fnbot.2014.00012

**Published:** 2014-03-20

**Authors:** Ritta Baddoura, Gentiane Venture

**Affiliations:** ^1^Inserm U846, Cortical Networks for Cognitive Interaction, Stem-Cell and Brain Research InstituteBron, France; ^2^GV Lab, Department of Mechanical Systems Engineering, Tokyo University of Agriculture and TechnologyTokyo, Japan

**Keywords:** social robotics, human-robot interaction, affective state, motion measures, mathematical modeling, assistive robot, familiar, fear

## Abstract

During an unannounced encounter between two humans and a proactive humanoid (NAO, Aldebaran Robotics), we study the dependencies between the human partners' affective experience (measured via the answers to a questionnaire) particularly regarding feeling familiar and feeling frightened, and their arm and head motion [frequency and smoothness using Inertial Measurement Units (IMU)]. NAO starts and ends its interaction with its partners by non-verbally greeting them hello (bowing) and goodbye (moving its arm). The robot is invested with a real and useful task to perform: handing each participant an envelope containing a questionnaire they need to answer. NAO's behavior varies from one partner to the other (Smooth with X vs. Resisting with Y). The results show high positive correlations between feeling familiar while interacting with the robot and: the frequency and smoothness of the human arm movement when waving back goodbye, as well as the smoothness of the head during the whole encounter. Results also show a negative dependency between feeling frightened and the frequency of the human arm movement when waving back goodbye. The principal component analysis (PCA) suggests that, in regards to the various motion measures examined in this paper, the head smoothness and the goodbye gesture frequency are the most reliable measures when it comes to considering the familiar experienced by the participants. The PCA also points out the irrelevance of the goodbye motion frequency when investigating the participants' experience of fear in its relation to their motion characteristics. The results are discussed in light of the major findings of studies on body movements and postures accompanying specific emotions.

## Introduction and theoretical background

Humanoid robots are expected to interact with humans in an efficient and natural way: intuitive and easy exchanges are the two main characteristics defining the sociability of artificial agents (Brayda and Chellali, 2012). Social interaction and robot acceptability are amongst the most important concepts explored in social robotics nowadays; acceptability or user acceptance being usually defined as the “demonstrable willingness within a user group to employ information technology for the task it is designed to support” (Dillon, 2001). Few human-human social interactions happen without experiencing some ambiguity or ambivalence, especially in a first encounter. When it comes to human-robot interaction (HRI), questions of social acceptance and of “intuitive” and ”successful” interactions seem more crucial since the difference between humans and robots is ontological.

The quality of such interactions depends strongly on the robot: not only on its appearance, but also on its abilities, features and autonomy degree. Most studies (e.g., Canamero, 2002; Bartneck et al., 2009) agree on the fact that further research is needed to better understand and determine which aspects and degrees of likeability and of similarity between humans and robots are required in order to enable more empathic and intuitive HRI. The interaction quality depends also on the humans' perception and appreciation of the robot and their readiness to adapt to it, thus taking its abilities and limitations into account and compensating for them in order for the interaction to happen (Takano et al., 2009). Although frequently used by the scientific community, expressions such as acceptance, and intuitive or natural interaction, refer in reality to psychological states and social codes difficult to precisely define (Turkle, 2006; Lee et al., 2011). In addition, as shown by many studies (Lee, 2008; Lee et al., 2011) the understanding of what makes an interaction succeed as well as be experienced as socially adapted and pleasant for humans, is generally exposed to interpersonal (Walters et al., 2008; Fischer, 2011) and intercultural variations (Fanaswala et al., 2011). Therefore, the variety in humans, but also in robots and in possible encounters, makes the study of HRI more complex and therefore more expressly needed.

To get a closer, more subjective, perspective on the human experience of encountering and interacting with a robot for the first time, we proposed to use the notion of the familiar considered here as an affective state. Therefore, we started exploring it in series of innovative studies (Baddoura et al., 2012; Baddoura and Venture, 2013a; Venture et al., 2013) with the aim to directly investigate the familiar and better define it in relation to other important concepts in HRI such as anthropomorphism, social acceptance, human readiness to interact with a robot or the robot's social skills. We believe that investigating the reliability of considering the familiar as an affective state is a challenging thesis that needs to be carefully explored and validated through different experimental stages in order to properly determine if the familiar can be legitimately considered as an affective state and as an operational concept to measure human emotional response. If validated, it would provide a comprehensive way to access the human experience during HRI and be an efficient indicator to evaluate the interaction's quality, as we believe it to have a major impact on its success. What we might gradually learn about the familiar state will probably enable robots to recognize in human partners states of familiarity or unfamiliarity, thus adapting their actions toward a more familiar presence. It will also support the design of robots that are able to reinforce a familiar state while interacting with humans.

When working on socially adapted robot behavior, numerous reviews in HRI use terms such as “to familiarize with,” “familiarity,” or “familiar,” all generally referring to what is known, well acquainted, often seen, or to what becomes known and usual due to repeated exposure and habituation. Nevertheless, we think that beyond its common uses as an adjective or verb to qualify what seems known or habitual, the familiar can be experienced as an affective state, related to specific emotions and thoughts. In our research, we are interested in the familiar as a state that may be experienced in a relatively limited time frame and may occur during new encounters, drawing possibly information from past experience but mainly forming itself in the present. This differs fundamentally from the “familiarity principle” also known as the “mere exposure effect” (Zajonc, 1968; Miller, 1976) which focuses on familiarity built up through repeated exposure.

Considered from a qualitative point of view, “the familiar” taken as an affective inner state is a notion that lacks definition and precision. However, it is possible from a quantitative point of view, to address it and measure it precisely by asking participants to assess how familiar they felt with a robot at different points of a first-time short interaction with it (using the Likert scale).

We do not know of prior studies directly addressing the familiar as an affective state, particularly in HRI. By connecting our experiment to other works that tackle ideas and concepts related in many ways to it, we aim at showing that HRI studies would clearly benefit from better understanding and defining the familiar. The recent years have showed an increasing interest in questioning human acceptance of robots. Nevertheless, the familiar as a topic has been cited or evoked in HRI studies mostly in indirect ways. For instance a study conducted on the implementation of a conversational robot in an elderly care center (Sabelli et al., 2011) showed that it is possible to help humans interact with the robot in a way that is familiar to them even though the robot itself was not familiar; the reference to the familiar here was not more explicitly explained or furthermore explored.

One source of inspiration for our work is the “Unheimlichkeitsgefühl” concept (Freud, 1919). Its translations vary: “the uncanny,” “feeling of strangeness,” “incredible familiarity.” The “Unheimlichkeitsgefühl” concept, together with Jentsch's elaboration of it (Jentsch, 1906), has inspired Mori's concept of the “Uncanny valley” (Mori, 1970). Beyond that, it is the subtle ambivalence it brings to what is known, new or acquainted that interests us. This association of the strange and the familiar can be useful when working on the interaction between a human and its artificial humanoid double.

In our first study on the familiar state (Baddoura et al., 2012), using the same experimental set-up and procedure described in this paper, but testing different hypotheses, we have shown the coexistence of moderate to high familiarity with feelings of strangeness. Results also showed that the familiar state can be experienced in a new and unexpected short interaction with a humanoid. These results underlined strong and positive correlations between experiencing familiarity and anthropomorphizing the robot, as well as perceiving the interaction as generally positive and pleasant, and more particularly as a comfortable, secure, safe, meaningful, and easy one. Further analysis showed (Venture et al., 2013) that the more humans experience the familiar while interacting with a robot, the more they are prone to react adequately to its engaging actions.

Though movement has been from the beginning a core theme in HRI, it is only with more recent studies that its social and psychological impacts (e.g., greeting gestures) have been directly targeted. It has been shown that embodied non-verbal interactions are fundamental to regulate human-human social interactions (e.g., Gillespie and Leffler, 1983). Non-verbal communication has the ability to replace verbal language to a large extent, especially when it is about communicating simple information, giving social cues or conveying emotions and intentions (Kanda et al., 2004). The importance of building communicative robots that are able to generate social cues through gesture has been shown by some recent studies (e.g., Kim et al., 2013). Kim et al. (2013) were also able to underline the positive effects of gestures during HRI by proving that people have a more meaningful social interaction with a robot and enjoy it more when the robot shows gestures than when it does not. Also, people will report a greater level of engagement with a robot when the robot shows gestures during an interaction than when it does not (Salem et al., 2011; Kim et al., 2013).

Simple social conventions such as daily greetings can have a strong and direct impact on the perceptions of others' feelings thus playing an important role in maintaining social ties. Sabelli et al. (2011) have shown that daily greetings performed by a robot in an elderly care center bring positive effects like pleasure and interest to the elderly. Our experimental situation begins and closes, as in real social situations, with welcoming and goodbye (non-verbal) gestures performed by the robot. In Baddoura et al. (2012) we showed that the social greetings performed by the robot, promote its polite and sociable character. We also proved for half our samples that the more intense was the familiar state experienced, the more sociable the robot was perceived (Baddoura et al., 2012), and the more humans understood its actions (Venture et al., 2013). In Baddoura and Venture (2013a) we also proved that experiencing a high level of familiarity when interacting with a robot strongly associates with high levels of adequate responses to its proactive useful-oriented actions, thus suggesting a possible impact of the familiar on the human readiness to successfully interact with a humanoid.

Emotions have a certain impact on body movements and postures of the person who is experiencing them. This effect is reflected in both internal physiological changes and external physical expressions (Frijda, 1986). Body movements and postures have received much less attention than changes in physical appearance (mainly in the face expressions) produced by emotional motor expressibility, the elements that have been more expressively considered are head movement and position, arms, legs and body posture (mainly when studying gait or locomotion) (Harrigan, 2005). Whether body movements and body postures indicate specific emotions is a matter of debate. While some studies have found evidence for specific body movements accompanying specific emotions, others show that movement behavior (aside from facial expression) may be only indicative of the quantity (intensity) of emotion (arousal perspective), but not of its quality (specificity perspective, Ekman and Friesen, 1967). Scherer and Wallbott (1990) also proved that movements could be influenced by the emotional state of a person. De Meijer (1989) showed that judges are able to infer specific emotions in recognition studies from body movements alone, which suggests that there are both body movements and postures which mostly reflect the activity level of emotions, as well as others that are associated in a distinctive way with specific emotions.

Even in the recent studies, few emotions have been widely analyzed; numerous studies focus on the basic emotions (Fear, along with anger, disgust, enjoyment, sadness and surprise). Wallbott (1998) in a study that became a reference and is still, worked on a large range of emotions including the fundamental ones, social ones and some discrete emotions. The main hypothesis of this work was that body movements and postures allow reliable distinction between emotions, thus reflecting not only the quantity of an emotion but its quality also. In general, the results of Wallbott's study were congruent with Darwin's study on body movements and postures accompanying specific emotions (Darwin, 1872/1965), and demonstrated that to some degree, certain body movements and postures are specific for certain emotions and that emotion was the predominant factor that determined movement and postural behavior of the actors in the study. More particularly, Wallbott's results indicated that movement and postural behavior are certainly indicative of the quantity (intensity) of various emotions, whereas certain distinctive features in movement and postural behavior seem to exist which allows the identification of certain specific emotions (quality).

Wallbott (1998) showed that when experiencing fear, the body had less movement activity: almost no lateral/fontal/sideways hand or arm movements; and the head moved rarely or almost not and the motion frequency was very low or close to zero. Camras et al. (1993) found that body activity accompanying discomfort was judged to be more jerky (less smooth) and active compared to that accompanying sadness. The negative segments were accompanied by relatively little body movement overall. Boone and Cunningham (2001) found that large and smooth movement was associated to pleasant or joyful emotion, whereas dreadful sorrowful emotion caused shrinking movement, and negative emotion brought jerky movements in uneven beats as well. When working on how motion characteristics affected perception of emotion during knocking and drinking arm movements, (Pollick et al., 2001) found fast and jerky movements to be more associated with anger and happiness, while slow, smooth movements were linked with sadness. Wang et al. (2012) showed that people have often violent head motion when expressing fear and confirmed that the information of head motion is mostly useful for spontaneous facial expression especially for distinguishing fear. The head movements have been largely regarded as an important element of the attitudes and the emotion states of a subject (Harrigan, 2005). If some of these movements, often spontaneous, are random and specific to each individual, some meaningful information for expression recognition have been extracted from the analysis of head motion features (Lv and Wang, 2010; Zhang et al., 2011).

When studying the gestural arm movement, it is mainly the amount of movement, the movement speed, the force, the fluency, the amplitude, and the height/vertical position that are addressed, but also rhythm, beat, sequence, leaning direction, tension flow, dynamic complexity, general spatial orientation (De Meijer, 1989; Dahl and Friberg, 2007). Castellano et al. (2007) showed the validity of using movement and gesture as reliable indicators of the state of individuals and Dael et al. (2013) confirmed the importance of arm movement in communicating major emotion dimensions (especially in multimodal non-verbal emotion communication): arousal and potency being much stronger determinants of the perception of gestural dynamics than the differences between positive or negative emotions.

In Baddoura and Venture (2013a,b), we started exploring the relations existing between motion and emotion. More precisely, we looked for dependencies between on one hand: the participants' evaluation of their affective states and their evaluation of the robot partner and of the interaction, and on the other hand: the motion data analysis of the spontaneous head and arm gestures of the participants in reaction to the robot, especially regarding the intensity of the movement, but also regarding its frequency and smoothness. For instance, Kanda et al. (2003) showed meaningful correlation between certain body movements (such as eye contact and synchronized arm movement) of humans interacting with a robot and their subjective evaluations of this robot, thus suggesting that humans make evaluations based on their body movements. In Baddoura and Venture (2013a), we were for example able to show that the more humans found the robot sociable, the more intense was their arm movement when responding to its social gestures. We also proved that when the robot is perceived as sociable, the faster is the participants' arm motion and the more adequate is their response to its engaging actions, whereas their head moves in a more brisk manner (Baddoura and Venture, 2013b). Finally, motion data showed that the more participants found the interaction familiar, the more they were close to the general tendency of the group: to react to NAO's gesture and take the envelope from it (Venture et al., 2013).

In the present paper, we focus on the participants' feeling familiar or frightened when interacting with the robot and the smoothness of their head motion measured all along the interaction. We also focus on their arm motion measured at two particular moments of the interaction, precisely when taking the envelope from NAO (smoothness) and when waving back goodbye (smoothness and frequency). To the best of our knowledge, no prior studies on the familiar as an affective state or category of emotion were done. As the familiar has been shown to be associated with positive emotions and mental states in our previous study (Baddoura et al., 2012), and as no existent studies give us reliable clear results to refer to, we based the formulation of our hypotheses on the main tendencies found when investigating the connection between body movements and emotions/affective states such as joy and pleasure, and in opposition to tendencies found between body movement and negative emotion such as discomfort. As for fear, we formulated our hypotheses with more confidence, in the continuity of the congruent results of studies such as the ones by Camras et al. (1993), Wallbott (1998), Boone and Cunningham (2001), Pollick et al. (2001), Harrigan (2005), Wang et al. (2012) as presented previously. Therefore, we hypothesized the following:

H1: The more intense the fear experienced by the participants during the interaction, the less smooth (the jerkier) is their head movement (a), the lower is the frequency of their arm motion when greeting goodbye (b), and the less smooth (c) is their arm motion when greeting goodbye, and when taking the envelope from the robot (d).
H2: The more intense the familiar experienced by the participants during the interaction, the smoother is their head movement (a), the higher is the frequency of their arm motion when greeting goodbye (b), the smoother (c) is their arm motion when greeting goodbye, and the smoother is their motion when taking the envelope from the robot (d).

## Materials and methods

The experiment involves a triad: a robot and two participants (X and Y) at a time. The participants are only invited to answer a questionnaire on the perception of robots. They are informed that the set is filmed and that sensors are placed around their head and wrist for motion capture. They do not know about the robot's intervention. The only instruction given to them is to answer a questionnaire. The experiment's scenario was validated by the Japanese ethical committee.

Participants were randomly assigned to one of the two sitting positions that resulted from a 1(X) × 1(Y) between-subjects design [NAO's behavior when handing the envelope: Smooth (with X) vs. Resisting (with Y)]. Resisting behavior refers to the fact that NAO stands slightly farther from Y than it did from X when handing him/her the envelope. It also refers to NAO keeping the envelope for 4 s in its fingers before releasing it to Y, whereas the release to X was immediate. Once the experience starts, there is no further intervention from the staff. Participants are not instructed about what they ought to do, it is all upon their own judgment. The scenario's duration involving the robot is about 1 min. The questionnaire requires 5–10 min to be filled.

### The robot

NAO (Aldebaran Robotics) is a 57-cm tall commercial humanoid robot. Its body has 25 degrees of freedom (DOF) whose key elements are electric motors and actuators. We used the programming software delivered with the robot to control it. We deliberately chose feed-forward control of the robot for repeatability. Of course, the substitution of NAO with any other robot can change the impression felt during the interaction, yet it would not change the association of certain physical behaviors (motion) with the mental and psychological states of the participants.

### The participants

The 20 pairs of students, 40 students in total (14 women, 26 men), were recruited on the campus of Tokyo University of Agriculture and Technology, and volunteered to participate in a study on the perception of robots. Participants range in age from 19 to 35 years (X: *M* = 23.75, *SD* = 3.53; Y: *M* = 22.7, *SD* = 1.68). Though previous exposure to robots was not controlled when recruiting them, candidates were mainly students from agriculture, biology and chemistry departments. We considered that having seen a robot in videos or having been exposed to a robot does not necessarily mean exposure to a humanoid robot or to the same robot used in the experiment. The interactive and relational dimensions involved in HRI are more subjective than rational and even a person who is used to manipulating robots might, once the robot manifests as an interaction partner, not behave with the same comfort than the one expected. Finally, the scenario of the interaction and its environment are most likely to be completely new to the participants.

### Experimental choices and set-up

**Figure 1:**
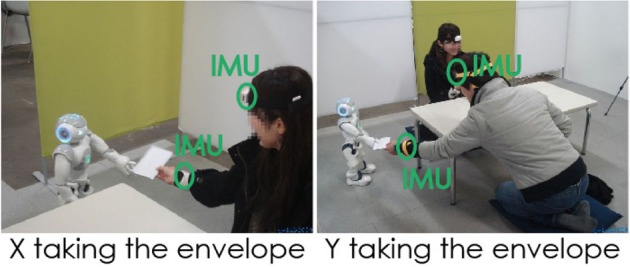
**The experimental set-up**. Participant X takes the envelope from NAO. Participant Y leans forward and extends his arm in order to grasp the envelope that NAO is handing him.

1. The set-up consists of a rectangular area limited by colored screens. It is furnished with a carpet, a low table equipped with pens, and two cushions put directly on the floor on each side of the table, providing therefore a comfortable Japanese-style ambiance, closer to a cozy space rather than to an anonymous lab. When seated on the cushions, participants are positioned on a low level which, given NAO's height, enables face-to-face contact.
2. The experiment starts with NAO entering the room, facing the table and holding in each hand an envelope with the word “Questionnaire” obviously written down on it. NAO walks toward the participants, then stops a few centimeters away from the table and greets them by bowing (its head bends with a slight forward bending of the upper torso). NAO turns toward participant X sitting to its left and extends its left arm holding the envelope in their direction. After a few seconds, its fingers release tension and the envelope is then ready to fall down in the participant's hand or on the floor, depending on the participant's reaction. Then NAO turns toward participant Y, extends its right arm holding the second envelope in their direction. NAO is slightly more distant from participant Y than it was from participant X; so in order for the envelope exchange to happen, Y has not only to extend his/her arm, but also to lean forward and reduce the distance from NAO (Figure 1). Another difference from the interaction with X is that NAO will now keep the envelope 4 s between its fingers before releasing it. Having delivered both envelopes, NAO waves goodbye with its right hand, turns around and walks back toward the door.
  Participants are free to start filling the questionnaire any time after receiving the envelope. We chose to ask them to answer the questionnaire at the end of the encounter so that the interaction stays uninterrupted, and to enable the candidates to remain as natural as possible without any disturbance. The whole situation lasts for 1 min only and the participants' memory and impressions about their encounter with the robot are likely to be still fresh.
3. Having two participants at a time might probably bring some uncontrolled variable, but it also contributes to limit the artificial dimension of the experiment and enhance the real dimension of the encounter. This choice allows NAO to manifest different -possibly perceived as “subjective”- behaviors regarding the same action: delivering the questionnaire. Furthermore, we felt that the stress that might generate from the unpredictable factors proper to the situation as well as from a close encounter with a robot might be counter-balanced, or at least eased, by being two persons facing the robot (all the pairs were recruited together and consequently knew each other).
4. The robot shows a slightly different behavior with participants X and participants Y, which allows limiting its repeatability and predictability (and mechanical functioning as a machine). It also allows seeing how this difference of behavior would be interpreted by the participants. This situation is a particular illustration of what could happen in public (or even domestic) spaces where the robot has precise tasks to accomplish and is prone to interact with different users that are not inevitably aware of its intervention and are relatively free to interact with it.
5. NAO is not presented here as an experimental object/tool but rather has a proactive role and a real essential task to accomplish which makes it a clear potential interaction partner. Furthermore, it punctuates the encounter's beginning and end with non-verbal greetings (NAO bows in the beginning according to the Japanese way of greeting and greets goodbye by waving its hand in a more international style this time).

### Data collection

We used in this study different but complementary tools in order to have a more accurate access on the participants' experience as well as to explore the possibility of combining variables of different kinds (e.g., answers to the questionnaire and reactions to the robots) to analyze the data available.

**Figure 2:**
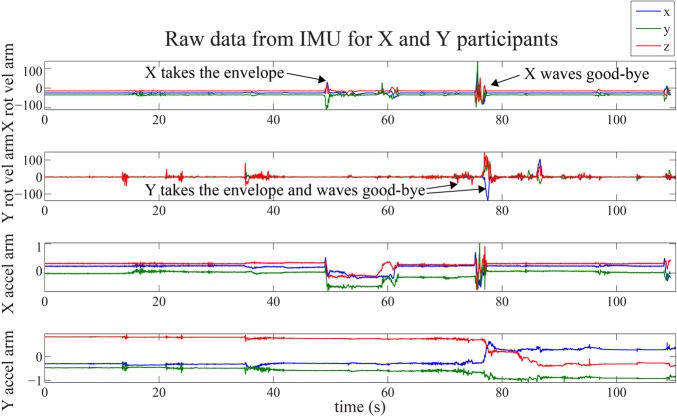
**Raw motion data collected with the IMU of the arm for both participants (rotational velocity in deg.s^−1^ and linear acceleration in m.s^−2^)**.

1. The questionnaire (Appendix A) consists of three parts addressing different topics but also sometimes the same topic considered from different perspectives. The questionnaire is written in Japanese to avoid possible confusions in the nuances that an insufficient level of English could bring. It consists of a first part using a 7-point Likert scale, a second part with Multiple Choice Questions, and a third very short part consisting of two open-ended questions enabling the participants to describe NAO and the interaction with it in their own words. In the present study we focus on the participants' ratings of their affective states (feeling familiar, feeling safe, or frightened) during the interaction with NAO. These ratings are obtained using the 7-point Likert scale: 1 meaning, e.g., “not feeling familiar at all,” 7 “Highly/intensely familiar.” We added 0 for “Irrelevant statement” to allow a more precise expression.
2. Each experimental session is video recorded using two stable cameras: one is filming the set from behind and gives images of the robot entering the set and of its interaction with the participants. The other is facing the participants and providing images of their movements and facial expressions. This tool is particularly used to collect data on the participants' non-verbal behavior and on their reactions (answer back or not) to NAO's gestures. The recorded data is reinforced with observation notes taken by the psychologist of our team.
3. Two IMU (Inertial Measurement Unit) are used for each participant. One is fixed on the forehead to capture the head and upper torso movements; the other on the arm -the right arm for X and the left for Y, each being respectively the closest arm to the robot's position and the one to be most likely used (from our observations on a pilot study of 20 candidates) by the participants to fetch the envelope. The IMU measure the longitudinal accelerations and the rotational velocities around 3-axes. Thus, more discrete micro-movement data is recorded giving another level of information regarding the participants' experience and reactions to NAO. Data for two pairs of candidates are unavailable. An example of raw data for the hand is given in Figure 2, for participants X and Y. The timing of the interaction with the robot is also specified.

### Data analysis

1. The motion data are analyzed from the IMU and from the video. From the IMU data, the three components of the rotational velocity and the three components of the acceleration are post-processed separately to obtain three types of information: motion intensity, frequency and smoothness. Only the last two are addressed in the present paper. The data recording starts a few seconds before the robot enters the room. The data are manually segmented using both the raw data collected by the IMU and the synchronized video data in order to refine the segment point. We distinguish several segments: (1) The whole interaction from when the robot enters the room up to when it leaves; (2) the grasping of the envelope starts when the arm movement is initiated and finishes once the envelope has been grasped, the participant's hand is not necessarily back to its original position; (3) the greetings movements are segmented from the initiation of the movement to the end of the waving, the participant's hand is not necessarily back to its original position. The frequency analysis and the jerk analysis are performed on each segment corresponding to the interaction with the robot (taking the envelope, waving goodbye) of the data, and during the entire interaction. Frequency analysis: First a simple frequency analysis (Fast Fourier Transform) on the hand motion data (angular velocity) is performed during the grasping motions and when answering the robot's goodbye by waving the hand; the frequency (*Hz*) of the first highest pick is used. Motion smoothness: it is computed during the overall interaction. There are several methods to assess the motion smoothness (Rohrer et al., 2002); we chose the jerk metrics (1/*s*^2^). For that, the accelerations are used to compute the jerk magnitude averaged over overall motion and normalized with respect to the peak speed. The smaller the jerk metric is the smoother the movement is.
2. We calculated the descriptive statistics (95% Confidence Interval) related to the participants' reactions to the robot's engaging actions to interact as well as the descriptive statistics related to their answers to specific parts of the Questionnaire related to the hypotheses addressed at each stage of our ongoing study. We calculated the Cronbach's α reliability of the first part of the questionnaire (the other parts are not addressed in this paper), whose answers were mainly used to investigate the participants' evaluation of the robot and of their interaction with it; knowing that this evaluation includes the participants' ratings of their affective experience. The calculated Cronbach's α indicates that the questionnaire is valid and has a good internal reliability (Table 1).

**Table 1:** **Cronbach's α reliability test for selected items in the questionnaire (Part I) related to the participants' general appreciation of NAO and of the interaction with it (A), the positive adjectives (B), and the negative adjectives (C) proposed to describe the participants' affective state and opinion about the interaction**.

**Evaluation of the encounter**	**(A) General evaluation**	**(B) Positive experience**	**(C) Negative experience**
Sections and items of the questionnaire (Part I)	A & B	B2, B3, B6, B7, B11, B12, B13	A1, A5, A10, A15, B5, B9, B17
Cronbach's α	0.83	0.79	0.81

## Main results

**Figure 3:**
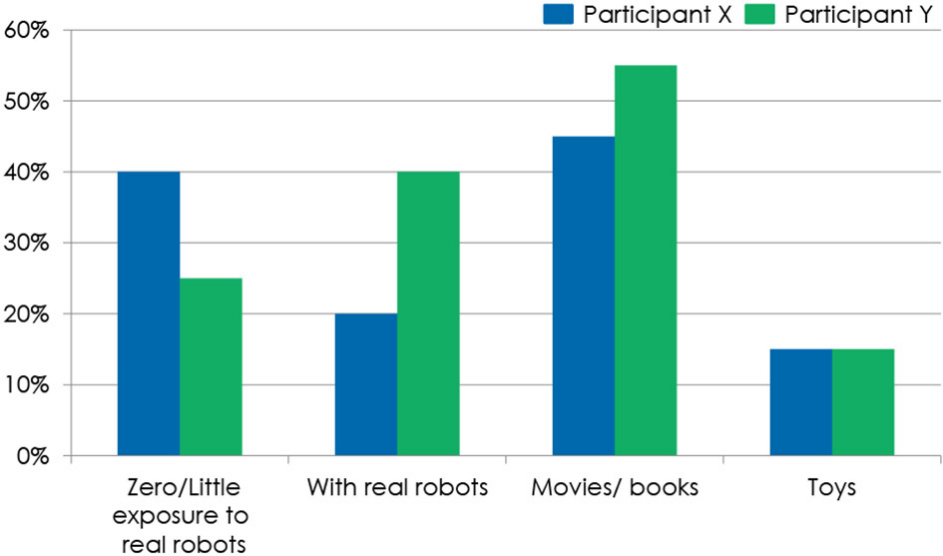
**The participants' earlier exposure to robots and source of exposure: movies and/or books, toys, real robots or no/little exposure**.

**Figure 4:**
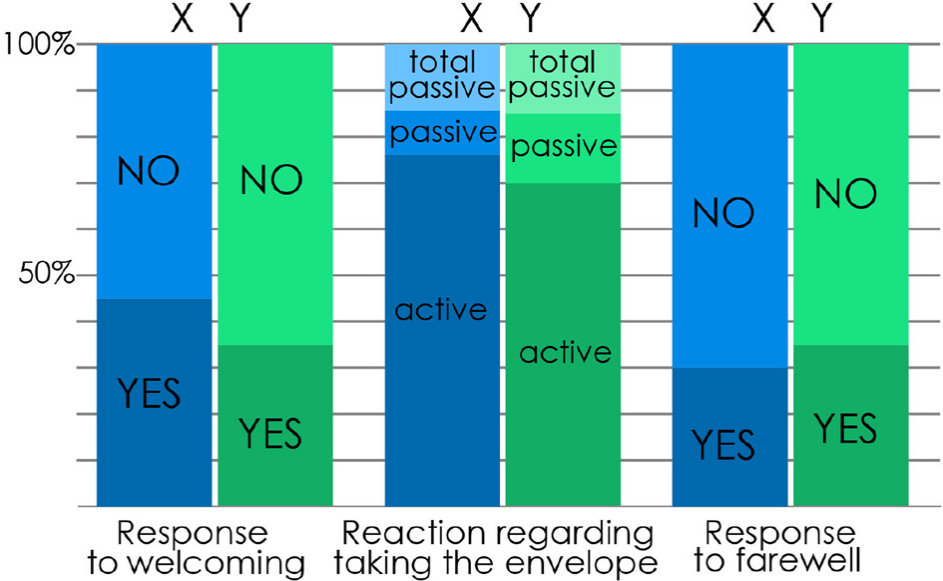
**The participants' response to the robot's gesture**. X participants' response is in blue and Y participants' response is in green. Response to welcoming: Participants' reactions to NAO's bowing to greet them hello (Yes: the participant answered back NAO's welcoming by bowing back; No: the participant did not react to NAO's welcoming). Reaction regarding taking the envelope: Participants' reactions to NAO's movement of extending its arm to hand them the envelope containing the questionnaire (Active: The participant extends his/her arm and leans forward if needed –for Y participants- in order to take the envelope; Passive: The participant does not react to NAO's attempt to hand her/him the envelope which falls down on the floor, but the participant takes it once it's on the ground; Total Passive: The participant does not react to NAO's attempt to hand her/him the envelope which falls down on the floor. The participant waits for the experimenter to come give them the envelope once the robot is gone and the encounter totally over). Response to farewell: Participants' reactions to NAO waving its hand to greet them goodbye (Yes: the participant waved back goodbye; No: the participant did not react to NAO's goodbye).

**Figure 5:**
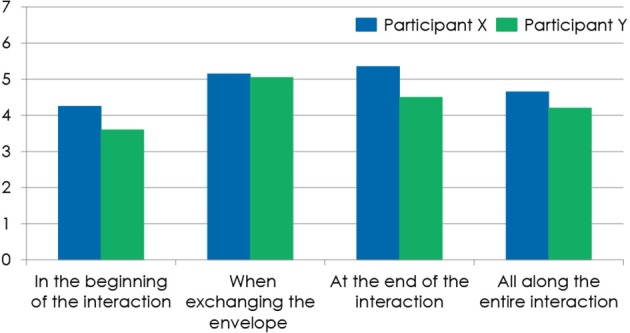
**The participants' experience of the familiar at the different key-moments of the interaction with the robot: in the beginning of the interaction, when taking the envelope from the robot, at the end of the interaction, and all along the entire interaction**.

**Figure 6:**
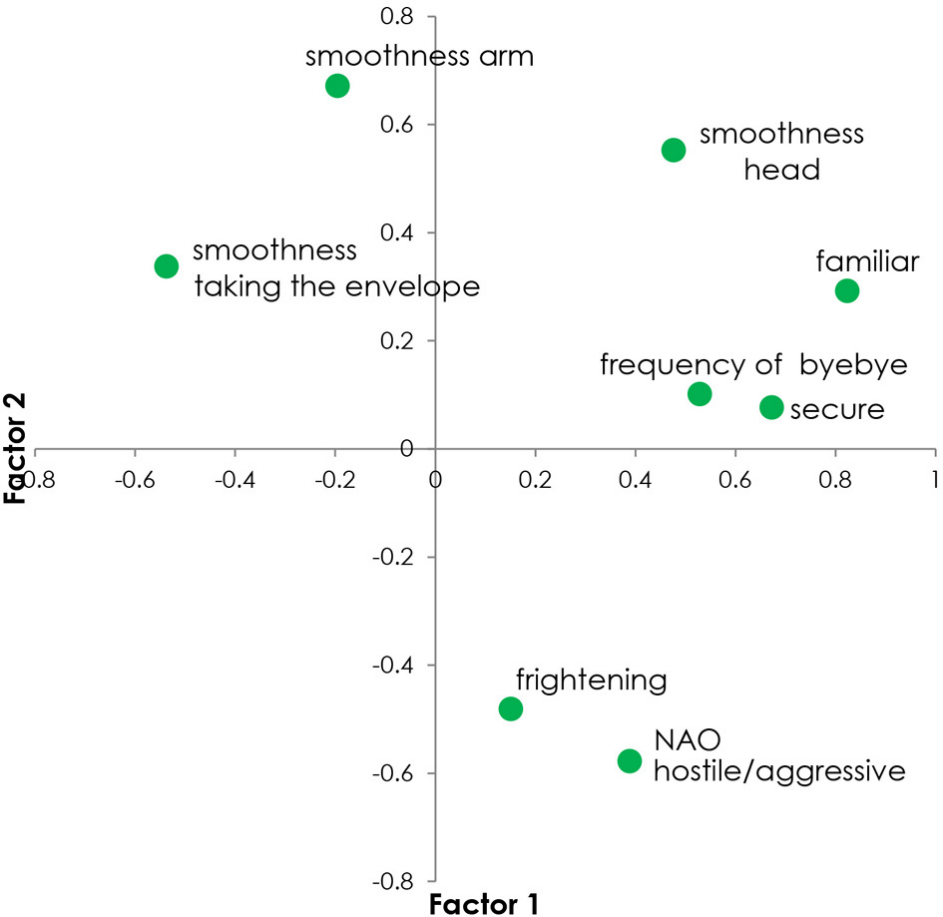
**Scatter plot (or Biplot) of the two first Principal Components (Factor 1 and Factor 2) of the Principal Component Analysis (PCA) of the affective/emotional variables and the motion variables examined in our hypotheses**.

Calculating the dependencies (Spearman's rank correlation coefficient) between different pairs of variables, particularly in relation to the participants affective states during the interaction (mainly feeling familiar, afraid, safe), and to their measured motion in response to the robot's actions, showed some interesting associations and also an absence of dependencies between certain pairs of variables (All significant and non-significant correlations are reported in Table A1, Appendix B). The principal component analysis (PCA) of our data revealed some significant information about the variables (Table 4, Figure 6). Only the most significant results related to this paper's hypotheses are presented here (Figures 3–6 and Tables 2–4). In Tables 2, 3, the *p*-value is obtained using the Student's t-distribution.

**Table 2:** **Spearman correlations between feeling familiar (F.) during the interaction with the robot and three motion measures**.

**Spearman correlation between F. and**	***p*-value**	**Corr**.
(A) Arm motion frequency when greeting NAO goodbye	0.01	0.569
(B) Arm motion smoothness when greeting NAO goodbye	0.01	0.653
(C) Smoothness of the head and torso	0.05	0.357
(D) Spearman correlation between S. and Smoothness of the head	0.05	0.388

The arm motion frequency when reacting to NAO greeting goodbye (A), the smoothness of the arm motion when reacting to NAO greeting goodbye (B), and the smoothness of the overall movements of the head and torso during the whole encounter (C). The correlation existing between feeling safe (S.) and the head motion smoothness is also reported (D).

**Table 3:** **Spearman correlations between feeling frightened (Fri.) during the interaction with the robot, and the arm motion frequency when reacting to NAO greeting goodbye**.

**Variables**	***p*-value**	**Corr**.
Fri. /Arm motion frequency when greeting NAO goodbye	0.05	−0.66

**Table 4:** **The two first principal components (PC) of the Principal Component Analysis (PCA) of the affective/emotional variables and the motion variables (Frq.: Frequency, Smo.: Smoothness) examined in our hypotheses**.

**Variable**	**PC 1**	**Variable**	**PC 2**
Familiar	0.8231	Smo. head	0.5530
Secure	0.6723	Smo. taking envelope	0.3376
Frq. Goodbye	0.5286	Familiar	0.2923
Smo. head	0.4763	Frq. Goodbye	0.1018
NAO hostile/aggressive	0.3882	Secure	0.0767
Frightened	0.1509	Frightened	−0.4810
Smo. taking envelope	−0.5372	NAO hostile/aggressive	−0.5774

### Earlier exposure to robots (Figure 3)

Earlier exposure was low for X (*M* = 2.55, *SD* = 2.06, s.e.m. = 0.42) and medium for Y (*M* = 3.85, *SD* = 2.43, s.e.m. = 0.54) (Baddoura et al., 2012). Generally, most of the participants (70% of the 40 participants) have never been exposed to a real robot before. *t*-test results showed that the difference between X and Y previous exposure to robots is not statistically significant (Venture et al., 2013). Also, most participants, whether having been previously exposed to robots or not, found interacting with NAO “New” (X: *M* = 5.6, *SD* = 2.0, s.e.m. = 0.4, Y: *M* = 5.1, *SD* = 1.7, s.e.m. = 0.4) (Baddoura et al., 2012).

### Reactions to NAO's gestures (Figure 4)

Most participants found rather easy/clear to understand NAO's actions (X: *M* = 5.25, *SD* = 1.71, s.e.m. = 0.38; Y: *M* = 4.8, *SD* = 1.73, s.e.m. = 0.38). Most participants (80% X; 75% Y) found it easy to react to it (Baddoura et al., 2012). Participants were mostly confused about taking decisions regarding: reacting or not to NAO's greetings and actions since they were not told too (35% X), opening or not the envelope (55% X, 35% Y) and taking or not the envelope when NAO resisted (55% Y) (Baddoura et al., 2012). Venture et al. (2013) data showed that the more participants found NAO's actions easy to understand, the more they were close to the general tendency of the group: react adequately to NAO's gesture and take the envelope. Also, the more the participants found no confusion in NAO's actions and found them easy to react to, the more intense was their motion when greeting it back goodbye (Venture et al., 2013).

Though they have not been previously informed about the interaction or instructed about what they ought to do, 80% X and 85% Y took the envelope from NAO (Figure 4). Of course, Y had seen NAO performing the same movement with X which might have facilitated their reaction, knowing that this possible effect was not addressed in our study. Nevertheless, as the novelty of NAO resisting before handing the envelope is introduced with Y, the large number of participants who adequately reacted is to be noted. Reacting to NAO's greetings was less effective as less than half of the participants answered to it (hello: 45% X; 35% Y; goodbye: 30% X; 35% Y) (Baddoura et al., 2012).

We ran a *t*-Test to compare X and Y participants' reactions to the robot's engaging actions. When comparing X participants' and Y participants' respective reactions to NAO's greetings, before (greeting hello) and after (greeting goodbye) exchanging the envelope, we found the difference in their response to be not statistically significant (Baddoura et al., 2012). The results of the *t*-Test showed similar lack of statistical significance when comparing between X participants' reactions and Y participants' reactions to NAO handing them the envelope. No statistical proof was found to assert that X and Y reacted differently to NAO, nor to assume that the difference of behavior showed by NAO when handing the envelope respectively to X and Y participants had a relevant impact on their respective reactions (to greetings and to the envelope exchange) (Baddoura et al., 2012).

### Feeling familiar during the interaction and its relation to motion (Table 2; Figure 5)

Most participants experienced medium-to-high familiarity while interacting with NAO (X: *M* = 4.9, *SD* = 2.0, s.e.m. = 0.4, Y: *M* = 4.9, *SD* = 1.6, s.e.m. = 0.4) (Baddoura et al., 2012). As for the different moments of the encounter, most participants reported feeling familiar with NAO all along the interaction with the highest scores for “the envelope exchange” moment and for the end of the encounter (Baddoura et al., 2012). 45% X and 50% Y found NAO familiar mostly from its behavior; 60% X found NAO familiar from the way it moves. 15% X and 10% Y found NAO not familiar at all (Baddoura et al., 2012). When comparing X participants' and Y participants' ratings of experiencing familiarity when NAO handed them the envelope as well as when NAO greeted them goodbye afterwards, the difference in their responses showed to be not statistically significant, thus due to chance (Baddoura and Venture, 2013a). *t*-Test results failed to validate the influence of NAO's changing behavior from X to Y on the intensity of the familiar state experienced by both participants (Baddoura and Venture, 2013a).

In this study, data analysis of the participants' movement during the envelope exchange showed a significant positive correlation between feeling familiar during the interaction with the robot and the frequency of the arm motion when greeting NAO goodbye (Table 2). Similarly, there is a strong positive correlation between feeling familiar and the smoothness of the arm motion when reacting to NAO's goodbye. The more the participants experience feeling familiar, the higher is the frequency of their arm motion and the smoother is this movement while greeting back NAO's goodbye.

When considering the head motion, its smoothness appears to be positively correlated with experiencing the interaction as familiar (Table 2). This means that the head movement tends to be smooth when the participants feel familiar when interacting with the robot. On the contrary, the head motion is jerkier when the participants experience low familiarity during the interaction. No dependency could be validated between experiencing the familiar and the smoothness of the arm motion when taking the envelope from NAO.

As reported in Table A1 (Appendix B), experiencing the familiar is highly and positively correlated with feeling safe (0.495; *p* = 0.00116) and with feeling secure (0.49; *p* = 0.00) during the interaction. In general, the participants experienced medium-to-high feelings of safety when interacting with NAO (X: *M* = 5.3, *SD* = 1.72, s.e.m. = 0.38, Y: *M* = 4.9, *SD* = 1.74, s.e.m. = 0.39). Feeling safe is also positively associated to the smoothness of the head (Table 2) which indicates that the smoother the head motion is, the safer and the more familiar the participants feel.

### Feeling frightened during the interaction and its relation to motion (Table 3)

Most participants experienced a low level of fear while interacting with NAO (X: *M* = 2.6, *SD* = 1.98, s.e.m. = 0.44, Y: *M* = 2.45, *SD* = 2.0, s.e.m. = 0.45). An interesting dependency was observed (−0.290) between feeling afraid and feeling familiar when NAO makes the action of handing the envelope to each participant. Nevertheless, its risk error (*p* = 0.069) being not confident enough, the negative correlation between feeling familiar and feeling frightened, can not be considered as fully significant.

Data analysis of the participants' movement (Table 3) showed a negative correlation between feeling frightened during the interaction with the robot and the frequency of the arm motion when greeting NAO goodbye. The more the participants experienced feeling frightened, the lower was their arm motion frequency when greeting NAO goodbye. No valid correlations were validated regarding experiencing fear and the smoothness of the head and of the arm when taking the envelope from NAO and when greeting it back goodbye.

### Feeling familiar and feeling frightened in their relation to motion: the principal component analysis results (Table 4; Figure 6)

The results of the PCA of the affective/emotional data (feeling familiar, feeling secure, feeling frightened) and of the motion data (frequency of the arm motion when waving goodbye, smoothness of the goodbye motion, smoothness of the arm all along the interaction, smoothness of the arm when taking the envelope, smoothness of the head all along the interaction) related to our two hypotheses, showed interesting results. Our aim was, not only to reduce possible data redundancy, but to also get an insight on the motion measures that might more accurately associate with (and infer) the affective state of the participants, particularly regarding feeling familiar and feeling afraid. Table 4 presents the first two components extracted from the PCA.

Based on the first principal component, it seems that the motion measures principally reflect the participants' experiencing the familiar during their encounter with NAO. The smoothness of the arm when taking the envelope, the goodbye frequency, as well as the smoothness of the head, seem to be the best motion measures (among the ones used in this paper) for discriminating the participants' feeling familiar and secure. The smoothness of the arm when taking the envelope has a negative projection on to the first component, which underlines the negative correlation between the arm smoothness when taking the envelope and feeling familiar and secure (the more familiar the participant feels, the jerkier is his/her arm movement when taking the envelope from NAO).

The second principal component gives information about the participants' feeling afraid when interacting with NAO and perceiving it as hostile/aggressive, more particularly about the motion measures that seem to be mainly associated (with a negative correlation) with this negative state. Indeed, Table 4 shows a contrast between experiencing high levels of fear and having a jerkier (less smooth) head motion and also in a less decisive way, a jerkier hand movement when taking the envelope from NAO. Nevertheless, as we observed in Baddoura et al. (2012) that the participants experienced low levels of fear and were very few to find NAO hostile/aggressive, the relevance of the second principal component is therefore weakened.

## Discussion

The motion data analysis, in relation to the movement's frequency and smoothness, gives an interesting insight on a dimension of the participants' reaction that is not controlled or voluntary for them. Results in Tables 2–4 show that certain body movements and gestures can be closely associated to affective states and that the arm and head motions of a human interacting with a humanoid partner are strongly correlated with emotional experience, which is congruent with major results from the field.

1. Greeting back goodbye: The higher the frequency of the human arm motion when greeting back NAO goodbye, the more the participants feel familiar (Table 2) during the interaction and the less they feel frightened (Table 3). The smoother the human arm motion is when greeting back NAO goodbye, the more familiar the participants feel during the encounter (Table 2). All these elements seem to indicate that feeling familiar during an interaction with a robot, associates with a vivid social involvement in responding to the robot's greetings.
2. Taking the envelope: No dependencies were found between the movement of taking the envelope from NAO and the participants' experience of fear or of the familiar.
3. The overall movements of the head and torso during the whole encounter: The more the participants' head and torso movements are smooth, the more the participants feel familiar and safe. This might indicate that feeling familiar and safe supports feeling secure and confident during the interaction, which translates in smooth and even head movements.

Getting back to our hypotheses, only one out of the four dependencies predicted in H1 was validated in compliance with the preexistent results in the field. It is important to keep in mind that the participants experienced low levels of fear during the interaction with the robot which might be the reason why most of the predictions hypothesized regarding the correlation between feeling afraid and body motion failed to be validated (The participants did not feel afraid enough, so that their body motion conveys or associates in a sufficient way with their emotion). Results showed that the more intense the fear experienced by the participants during the interaction, the lower is the frequency (b) of their arm motion when greeting goodbye. However, H1 remains mainly non-validated, as our results failed to find any valid correlation between experiencing fear and the smoothness of (a) the participants' head movement and (c) of their arm motion when greeting NAO goodbye, and (d) when taking the envelope from it. When reading these findings in light of the PCA results, it appears that the arm frequency is not an accurate discriminatory factor to inform us about the fear experienced by the participants during this interaction. The validated dependency (H1/b) thus loses the strength of its significance.

As for H2, the results succeeded in validating far more correlations than for H1 (three out of four), and in conforming to the expected dependencies. Indeed, the positive dependency between the intensity of the familiar experienced during the interaction and (b) the frequency of the arm when waving back goodbye, (a) the smoothness of the head and (c) of the arm motion when greeting goodbye, were all showed. The more the participants felt familiar while interacting with the robot, the smoother their head motion was all along the interaction, and the faster and smoother was their arm motion when greeting back NAO goodbye. This finding indicates that when it comes to motion smoothness and frequency, relating the familiar state to emotions and affective states such as joy and pleasure, was accurate.

As for the arm motion smoothness when taking the envelope (d), the results failed to validate any correlation between it and the experienced familiarity. One possible explanation for that would be that the goal-oriented gesture of taking the envelope might not be familiar-specific, meaning that it might not be ideal or accurate to test the specificity of motion when experiencing the familiar. Indeed, some motion-gestures are more emotion-sensitive than others. Another interpretation would be that feeling familiar essentially, but also feeling safe (and in a way not feeling worried, insecure or frightened) during an interaction with a robot, would be possibly more important to take into account (thus being better encoded on the motion level) when the interaction is solely social than when it consists of a practical action. More generally, the social gestures of greeting hello and goodbye are considered as symbolic gestures, their goal being to induce expressive marking and communicate affective states (Nehaniv, 2005). This might explain why it was more possible to validate dependencies between the emotional dimension and the motion characteristics when greeting back NAO goodbye, than when taking the envelope from it, which is only a goal-oriented response to NAO' gesture of handing the envelope.

Also, in our study, the participants were students experiencing an announced interaction with a robot and not professional actors who were instructed to act certain emotions (which frequently leads to explicitly affective gestures that are acted in an exaggerated way, as underlined in many studies: De Meijer (1989), Boone and Cunningham (1998), Wallbott (1998), Atkinson et al. (2004). Furthermore, the affective states that the participants rated were spontaneously experienced by them and mainly triggered by the robot's engaging actions to interact (there were no forced-choice paradigms limited to the target emotions themselves; a variety of affective and mental states as well as of adjectives was proposed to the participants in the questionnaire -Appendix A- the robot gave them). All this might have had an impact on our results, especially regarding the absence of most of the expected correlations when feeling afraid.

More importantly, the PCA results strongly suggest that the head smoothness and the arm frequency when waving goodbye are the most reliable motion measures (among the ones used in this paper) to seriously consider, when exploring the participants' experience of the familiar in this study. Hence, what is truly validated in H2, and more generally in this paper, is that the more familiar the participants feel when interacting with NAO, the smoother is their head motion all along the encounter and the higher is their arm motion frequency when waving goodbye.

Another specificity of this paper is that the fear experienced by some of the participants is a “discreet” (rarely explicit), not elated, emotion. Fear is not experienced here to the extent of an emergency situation. In addition, the participants gave it low ratings, which might explain the motion measures' limitations when it comes to being fear-sensitive. It is also interesting to remember that, though the risk error was not confident enough to fully validate it, a negative correlation was observed on one hand between feeling afraid and feeling familiar. On another hand, feeling familiar was positively and strongly correlated to feeling safe and feeling secure. These findings suggest that experiencing a familiar state is not compatible with being afraid, or in other words: feeling familiar implies a low level of fear or an absence of fear, as well as a sufficient feeling of safety. These specificities are to be tested in future studies in order to improve what we already succeeded to show about the familiar, especially in regards to the fact that, as previously cited, (Baddoura et al., 2012) underlined the familiar's association with mainly positive affective states and experiences such as pleasure, security, comfort, (whereas most of the negative adjectives proposed to describe NAO and the interaction with it have received low ratings).

In a more general way, the absence of previous work to precisely hold on to when it comes to the familiar, makes it more difficult, but also more motivating, to make the right assumptions thus opening up to more trials and errors. It is also important to keep in mind that individual differences, an aspect that was not addressed in our analysis, especially in regards to temperament and arousal, but also NAO's special features (which make it probably less scary than bigger or more mechanical or even more human-like humanoids), might also have played a significant part when it comes to the lack of valid associations between feeling frightened and the smoothness of the head, the smoothness of the arm when waving goodbye and the smoothness of the arm when taking the envelope from NAO.

The dependencies existing between body motion and affective states seem once more decisive for the quality of HRI, as our results comply with the ones from many previous studies showing that the human affective experience can be readable from the motion measure and analysis. No definitive conclusions can be drawn from these results but some trends to be furthermore explored have emerged, especially regarding the familiar. We plan on working on further investigations with more accurate sets of gestures and of motion features, analyzed with a more developed statistical approach, our present paper being a work in progress. Indeed, other motion gestures and interaction scenarios, as well as other motion-cues not assessed here (e.g., speed, amplitude) might be better predictors of feeling familiar, feeling safe, or feeling frightened.

The associations validated in this paper between feeling familiar and some of the arm and head motion, support the fact that the familiar sketches a stimulating path for studying the affective experience as well as the body motion of a human interacting with a robot. However, the results obtained so far are not sufficient enough to precisely define the familiar and to fully assert that it ought to be considered as an affective state. Some tendencies and some associations have been brought to light and are to be completed with new findings. Once the legitimacy and the usefulness of considering the familiar as an affective state, are truly validated by a series of experiments, we will make a step further toward theorizing it and defining it in regards to the existing emotion categories.

We deeply believe that the familiar could give an interesting, complex and discreet access to the human internal experience during social interactions in general, and during HRI in particular, thus enabling us to assess through one single affective state: the familiar, various essentials such as the comfort, the pleasure, the safety, the interest, the easiness, and the success of an interaction. If proven useful and valid as an affective state, the familiar could contribute to enhance the development of emotional maps of social interactions and of body motion when it comes to designing social robots that are more biologically inspired (the robot internally simulates social processes) than functionally inspired (as it is the case in our study: the robot gives the impression of being socially competent without having an internal design based on nature) (Fong et al., 2003). In a future research, we make it a priority to improve what we started understanding about feeling familiar during an interaction, thus making a step further toward attempting to categorize the familiar as an affective state (especially in relation to self-conscious emotions, positive emotions, and pro-social emotions).

## Limitations

A pilot study with a human instead of a robot, as well as using the “earlier exposure to robots” as a controlled variable, might allow us to get more accurate results in a future research. We are aware that our results are limited to a specific interaction with a specific robot, NAO. Its particular design features seem to facilitate human acceptance and appreciation (most of the participants have reported finding NAO cute and seductive Baddoura et al., 2012). Conducting the same study with a different robot (e.g., bigger, mechanical appearance) would also be of interest. The study being conducted in Japan, our findings will gain to be compared to results from a future experiment conducted, e.g., in a western culture, in order to get an insight on intercultural variations. In that case, some experimental choices such as the Japanese design of the set-up, and the use of bowing by way of greeting, would be adapted to the experiment's social codes, in order to avoid cross-cultural bias.

Also, as already mentioned, taking into account the interpersonal variations would enable us to have more precise results. No attempt was done in this paper to study the possible effect of the individual differences on the results. As underlined by Bernhardt (2010) who is aiming at modeling individual differences, hence improving the accuracy of emotion recognition, “ignoring individuality of expression could limit the studies' applicability and real-world success.” Last but not least, the use of a broader multivariate statistical approach would strengthen the significance of our results and improve their accuracy.

## Author contributions

Gentiane Venture and Ritta Baddoura have equally contributed to this work (conception and design of the experiment, contribution of materials and analysis tools, data analysis and interpretation). Gentiane Venture has primarily developed the robot's motion design and has carried out the human motion capture and analysis. Ritta Baddoura has primarily developed the familiar as an affective state and has worked on the psychological aspects of the study.

### Conflict of interest statement

The authors declare that the research was conducted in the absence of any commercial or financial relationships that could be construed as a potential conflict of interest.

## Appendix A

Bilingual version of the questionnaire (the one used is only the Japanese version)

*Questionnaire*  Age ……            Gender M F

I- SCORE SHEET *0 – 7 scale*

Please circle the relevant score from 1 to 7 (1: Minimal intensity, 7: Maximum intensity)

If the answer for the question is irrelevant, please check 0.

III- OPEN QUESTIONS

A-How would you personally describe NAO in a few words?

B-How would you personally describe your interaction with NAO in a few words?

## Appendix B

**Table A1:** **Spearman valid and invalid correlations between various affective/emotional states rated by the participants, the participants' evaluation of NAO as possibly hostile/aggressive, and various motion variables**.

		**Smo. Goodbye**	**Smo. head**	**Smo. arm**	**Smo. taking envelope**	**Familiar**	**Fri**.	**Secure**	**NAO hostile**	**Safe**
**Frq. bye-bye**	Corr.	−0.44	0.06	−0.08	−0.05	0.57	−0.66	0.17	0.05	0.132
	*p*-value	0.30	0.74	0.68	0.81	0.01	0.05	0.36	0.77	0.478
**Smo. Goodbye**	Corr.		−0.47	0.74	−0.27	0.65	−0.56	−0.35	−0.65	−0.431
	*p*-value		0.26	0.04	0.55	0.01	0.15	0.39	0.10	0.334
**Smo. head**	Corr.			0.24	−0.04	0.36	−0.06	0.20	0.06	0.388
	*p*-value			0.16	0.83	0.04	0.73	0.26	0.75	0.0235
**Smo. arm**	Corr.				0.20	0.05	−0.05	−0.09	−0.16	0.12
	*p*-value				0.27	0.79	0.76	0.60	0.38	0.5
**Smo. taking envelope**	Corr.					−0.26	−0.149	−0.10	−0.06	−0.136
	*p*-value					0.14	0.399	0.58	0.73	0.445
**Familiar**	Corr.						−0.29	0.49	−0.17	0.495
	*p*-value						0.07	0.00	0.28	0.00116
**Fri**.	Corr.							−0.03	0.27	−0.218
	*p*-value							0.85	0.09	0.176
**Secure**	Corr.								0.17	0.243
	*p*-value								0.28	0.131
**NAO hostile**	Corr.									−0.211
	*p*-value									0.191

The affective/emotional variables are: feeling familiar, feeling frightened (Fri.), feeling safe and feeling secure during the interaction. The motion variables are: arm frequency (Frq.) when waving goodbye, arm motion smoothness (Smo.) when waving goodbye, arm motion Smo. when taking the envelope from NAO, Smo. of the arm all along the interaction and Smo. of the head all along the interaction.
